# Liminality and head and neck cancer: core concepts and applications for clinical practice

**DOI:** 10.3332/ecancer.2019.986

**Published:** 2019-12-12

**Authors:** Camilla Dawson, Jo Adams, Deborah Fenlon

**Affiliations:** 1 Queen Elizabeth Hospital Birmingham, Birmingham B15 2TH, UK; 2 University of Southampton, Southampton B15 2TH, UK; 3 University of Swansea, Swansea SA2 8PP, UK

**Keywords:** cancer, liminality, surgery, head and neck, neoplasm, survivorship

## Abstract

This article explores the concept of liminality and its potential application to understand the experience of being diagnosed and receiving treatment for head and neck cancer.The article describes how we identified liminality in people who had received surgical treatment of head and neck cancer and why it is important that the clinical team understand and consider liminality in their interventions, to improve outcomes.

**Background:**

Head and neck cancer is a disease that may affect the way an individual looks and functions, specifically with regard to eating and drinking. This qualitative study identified the perceptions of people who had been diagnosed with head and neck cancer and had undergone surgical treatment, to develop our understanding of what happens to people beyond their physical recovery in hospital.

**Methods:**

We carried out 1:1 face-to-face interviews with people in the acute phase of recovery following head and neck cancer surgery. We analysed and verified themes from the data, using an interpretive phenomenological methodology.

**Results:**

The study identified a liminal phase which people described during their recovery from head and neck cancer surgery. Individuals described no longer being the people they were before surgery, but not yet the people they would be. We explored this concept and suggest ways in which the clinical team may identify and explore liminality post head and neck surgery. This information may help clinical teams to support people manage complex issues related to the sense of self, identity and recovery after head and neck cancer surgery.

## Background

People who undergo surgery for treatment of head and neck cancer experience complex symptoms, including alterations to the way they function such as eating, drinking and the way they present themselves to the world [1]. The impact of diagnosis and treatment requires specific and targeted treatments from the multi-disciplinary team (MDT) including the surgeon, speech and language therapist (SLT), clinical nurse specialist and dietitian [2]. Although research has improved fundamental facets of recovery following head and neck cancer such as rates of survival following oncological treatments [3], quality of life [4] and physical rehabilitation strategies [5], there is limited qualitative evidence to improve how we deliver these treatments, specifically when the individual is in the acute post-surgical phase. We undertook this research to understand what the individual with cancer needed after surgery, and how they felt the clinical team could provide this care.

## Methods

We explored people’s experiences of the surgical treatment head and neck cancer using a phenomenological methodology, as described in other publications [6]. One-to-one interviews were undertaken with individuals who had head and neck cancer surgery. Inclusion criteria were any person having reconstructive surgery and swallow therapy for the treatment of head and neck cancer at the study centre with reconstruction using free, pedicle or composite flap, over 18 years old, capacity to give consent, English speaking. People who had undergone total laryngectomy were excluded from the study as they received specific swallow and voice rehabilitation as a result of having their larynx removed. Written consent was obtained from the participants before inclusion in the study, and full ethical approval was granted from the University of Southampton Ethics and Research Governance Online (reference: 12133) and the NHS Integrated Research Application System (reference: 164120).

Participants were interviewed while inpatients in hospital, between days 7 and 14 post surgery. Data were audio recorded by the author and transcribed verbatim. Inductive analysis was undertaken and was coded line by line, phrase by phrase, to the smallest meaningful component. Main themes, subthemes and categories were identified, defined and verified by the research team.

## Results

Several themes and subthemes emerged which broadly involved the individual’s inability to prepare for the enormity of the surgery, and the importance of feeling valued and acknowledged by effective communicative experiences with the clinical team [6]. However, this paper explores a liminality subtheme that underpinned all facets of recovery.

We identified liminality through the phrases people used to describe their time following surgery and their sense of transition, where they were no longer the person they were, but not yet recovered to the person they would be. Although this particular study did not explore the time beyond discharge from the hospital, it seems likely that the liminal phase continued beyond discharge and recovery at home. The liminal space people experienced during their stay in the hospital was a safe space where they could begin the process of adjusting to the changes that were happening to them before they had to go back to being in the world again. This transitionary phase happened in the ward while they were recovering from their surgery, receiving rehabilitation, before discharge home. Participants were no longer physically the people they had been prior to surgery, who were in control of their bodies and were not yet able to easily control their new selves. This was obvious in the challenges people experienced, predominantly with the way they looked, eating, drinking and speaking.

*‘I was frightened in case I couldn’t talk or make myself understood and I was worried in case I might be like that forever’* (Sandra line 37).

Sandra’s comment denotes inherent issues with communication following surgery, and the potential impact of this compromise long term.

*‘I am not going to want to go out and see people if I’m going to look like this for the rest of my life’* (Emma- line 131)’.

Participants reflected on the time from their diagnosis to treatment, and the transition they experienced.

*‘I said when I came in, I came in on the dark side and I’m coming out on the light side, and it’s happened’* (Annie line 150).

*‘I feel fine at the moment, when I get home and all that, get back to a routine and then get myself together, but at the moment I’m in a good place, I’m not in a bad place’* (Steve-369).

Participants described how the ward felt like a safe space following their head and neck cancer surgery, a sense of protection, understanding and acceptance seemed tacit components of this transitionary period.

*‘I’m not going downstairs ‘cause I’m embarrassed at the moment about the way my face is, but I’m stopping, being on the ward, but I’m alright’* (Steve line 178).

The data we describe identify a separation from ‘normal life’. The symptoms people experienced were grounded in their innate human functions, the way they communicated, ate and drank. Their functional compromise existed in the departure from their life pre-diagnosis, and stayed with them as they entered in to their next phase of recovery, accompanied by their rehabilitation.

## Discussion

Liminality is a concept explored within numerous spheres. In philosophy, the idea of a limit or boundary was considered by Plato referring to logic and sense-making, and latterly by Kant who dealt with what the limit represented or confined, and indeed what happened at the limit [7]. In anthropological terms, Gennep [8] first described liminality with regard to rituals, the cultural celebrations of the movement and transition from one social or religious state to another. These transitions could include rites of passage such as birth, coming of age, marriage and death. Rites of passage are related to these transitions where an individual severs social and cultural relationships, enters into a transient ‘in-between phase’ and then takes up a new social and cultural position [9]. Gennep (1960) explains transitions as implicit in our lives, forming a succession of common stages. The outcomes of the transition may be unknown and ambiguous, and liminality refers to the transition in itself. The concept of being on a threshold was further developed by Turner [10], who used the phrase ‘betwixt and between’. Since the advent of liminality, many sociological and anthropological frameworks have developed the concept, including health research.

Liminality has been identified in people’s experience of many different diagnoses and treatments of cancer [11]. The impact of a diagnosis of cancer on the individual is complex and unique, effecting physical emotional and psychological elements of self [12]. Little *et al* [11] suggested that as the body is the place that both the self and disease exist, when fundamental changes such as surgical or oncological treatments take place, not only the disease but also the self may also be altered. Little *et al* [11] offered a specific interpretation of liminality, developing components of the Gennep [13] theory. First, Little *et al* [11] explained that the liminal process is not easy to identify or define specific to one moment alone. Second, liminality is not static, but may continue for an indeterminate length of time where there may be an acute and chronic liminal phase, at diagnosis and beyond.

Liminality has been described in the experience of treatment of early breast cancer [14], prostate cancer [15] and chronic pain [16] among other diseases. The biographical disruption and transition which accompany liminality are also evident in other phenomenological works, such as Kalanithi’s [20] description of his diagnosis, treatment and palliative phase of lung disease. Kalanithi transitioned from a position of employment as a doctor with autonomy and choice to being a recipient of cancer treatment as a patient, to being cared for by his wife and family, and finally his breath became air as per the title of the book. This place between opposites [13] is an important facet of liminality. Whilst the nature of the transition may be difficult to describe, its contextual position and orientation to other social or cultural thresholds defines it. It is the movement between in and out of.

In the context of head and neck cancer surgery, it seems likely the individual’s successful ‘re-entry’ [9] into a meaningful social and cultural role could be influenced by careful and skilful navigation with the clinical team through this liminal state. As rehabilitation post-surgery is a central part of physical recovery, it is imperative that teams recognise and attend to these complex wider contexts. People are likely to require more than improvement in speech or swallow function alone. They may require recognition of the journey they have undergone, acknowledgement of the person who has been lost following surgery and support to re-enter their lives in a way they deem adequate, sufficient and meaningful.

It is also important to consider to what extent this liminal phase may be augmented by the clinical team. Whether the team can in fact influence the liminal phase, or whether the team become the ‘masters of ceremonies’ described by Gennep [13], supporting people to leave and re-enter society following a physical and emotional transition. When people are forced to face their limits, there is a significant responsibility for their emotional wellbeing and care. It seems unlikely that this need will be met if the fragility of the transition and transformation individuals experience after head and neck cancer treatment is overlooked.

The data we gathered suggested that the team could ease the exit and re-entry following the transformative experience, and provide reassurance and therapeutic interventions while the individual was ‘betwixt and between’. This could be achieved by clinicians recognising the existence of these wider issues, giving space for the individual to explore them and being prepared to allow the individual to describe what may help them achieve a new and meaningful life. The nature of a rehabilitative intervention to improve a function that is lost or damaged, during a time when a person is also experiencing an existential transition, requires careful planning and administration. The loss and change experienced was not limited to physical ability; therefore, rehabilitation should not solely address the physical issues. This concept fits with other themes in this research, where individuals needed to connect with the healthcare professional and experience space and support within the interventions they received.

It is a challenging time to discuss ways the quality of clinical interventions may be improved on a backdrop of political uncertainty regarding healthcare funding and increasing pressures to reduce bed stay days [17]. However, the findings from this study should not be overlooked; they identify the value of time spent on the specialist head and neck cancer ward after surgery where staff have the capability to create a physical and metaphorical space in which people are able to experience liminality, changes to the way they could speak and swallow and overwhelming emotions regarding loss, anxiety, change and recovery. This environment itself may be healing and therapeutic to be within, which should be considered and recognised, as bed stay days are seldom described in terms related to benefit to individuals. While it is not suggested that there should be extra time spent in the hospital when resources are limited, it is important that the true value and potentially invisible support provided by being within an institution is recognised. People were made to feel safe with the support of skilled professionals. Beyond this, it was clear that people could note and describe the importance of being with staff who helped them cope with their altered selves.

## Applications

It can be challenging to apply concepts about transition and transformation into every day clinical practice. Within the field of head and neck cancer, we are adept at measuring the quality of life and level of compromise, but translating these concepts into useable clinical skills is more challenging. It is important that we attempt to move beyond identifying what people cannot do, and the effect this has on their lives, to developing ways we can improve the way people live beyond cancer. At the study centre, we have started to provide swallow therapy to include eating and drinking in a public dining area, outside of the ward, with the SLT. This has been developed to address the social issues people described with looking and feeling different in a public arena. Moving the swallow therapy from the confines of the ward and applying the principles into everyday life seems to support the individual to feel safe and capable within a public environment. This also seems to reduce the individual’s sense of anxiety about participating in public interactions following discharge home. This intervention requires development and testing, to establish whether the departure from usual care improves outcomes, and if so how. This is being developed with patient groups and clinicians to evaluate this complex intervention.

### Rehabilitation strategy

The findings also suggest that rehabilitation interventions may need to include discussing transition, change and being on the threshold of a new physical and social sense of self. This may support the individual to experience these phenomena within a safe and supportive framework, while being enabled to communicate, swallow and function in the best ways possible. In practical terms, this could involve discussing how the individual may manage meal times at home, how they feel about going outside or to public environments and providing strategies to manage concerns about public eating and speaking. The importance of the hospital environment in which people receive care should be recognised and efforts should be spent protecting its rehabilitative value. It seems likely that offering therapeutic intervention outside of the hospital as an outpatient, to support this potentially ongoing journey is also appropriate.

### Intersectionality

In order to provide care that meets the holistic and complex needs of the individual, it may be of benefit for teams to consider an intersectional approach, which recognises the many components of an individual and the changes to their identity and sense of self after surgery. It seems possible that intersectionality may be appropriately translated to the post-surgical phase where people are simultaneously a patient, a parent, a member of society with a job and an altered version of all these ‘selves’ following surgery. Intersectionality covers the multiple categorisations of an individual; in feminist research, this tends to include race, gender and sexual orientation. Within feminist methodologies, intersectionality is concerned with issues associated with reducing a person to just one category, overlooking the relational element of social and cultural roles [18]. This feminist position on intersectionality provides a perspective in which a person is not reduced to ‘a patient’, and instead recognised with their plural identities. The adoption of this concept may limit the potential for a patient’s voice to be silenced by a professional [19], by enabling the individual and their concurrent and multiple emotional and physical needs to be heard and attended to.

## Conclusion

The liminal space was an important facet of the post-surgical experience. When the individual was on the threshold of the new self, they required space to exist as an altered person. This space was created in part through recognition of the social, emotional and physical loss of the individual that used to be. Social communication, physical rehabilitation of swallow and acknowledgement of the enormity of the emotional cost of surgery with the multi-disciplinary team were also healing. These concepts may be used by healthcare professionals across disciplines to shape interventions, influence therapy and to help individuals make sense of the complex impact of head and neck cancer surgery. This insight has the potential to improve ways we may support the person with head and neck cancer and help them adapt to life beyond surgical treatment.

## Conflicts of interest

The authors declare that they have no conflicts of interest.

## Funding declaration

The authors received no funding for this work.
